# Improvement of anti-cancer drug efficacy via thermosensitive hydrogel in peritoneal carcinomatosis in gastric cancer

**DOI:** 10.18632/oncotarget.22312

**Published:** 2017-11-06

**Authors:** Tae-Su Han, Keun Hur, Boram Choi, Ji-Yeon Lee, Sun-Ju Byeon, Jimin Min, Jieun Yu, Jung-Kyo Cho, Jimin Hong, Hyuk-Joon Lee, Seong-Ho Kong, Woo-Ho Kim, Kazuyoshi Yanagihara, Soo-Chang Song, Han-Kwang Yang

**Affiliations:** ^1^ Cancer Research Institute, Seoul National University College of Medicine, Seoul, Korea; ^2^ Department of Pathology, Seoul National University College of Medicine, Seoul, Korea; ^3^ Center for Biomaterials, Korea Institute of Science and Technology, Seoul, Korea; ^4^ Department of Surgery, Seoul National University College of Medicine, Seoul, Korea; ^5^ Department of Biochemistry and Cell Biology, Cell and Matrix Research Institute, School of Medicine, Kyungpook National University, Daegu, Korea; ^6^ Biotherapeutics Translational Research Center, Korea Research Institute of Bioscience and Biotechnology, Daejeon, Korea; ^7^ Division of Biomarker Discovery, Exploratory Oncology Research & Clinical Trial Center, National Cancer Center, Tokyo, Japan; ^8^ ezlab, Suwon, Korea

**Keywords:** gastric cancer, peritoneal carcinomatosis, PPZ hydrogel, docetaxel, live imaging

## Abstract

Peritoneal carcinomatosis (PC) of gastric origin has a poor prognosis with short survival due to lack of effective therapeutic modalities. Here, we evaluated the therapeutic efficacy of an injectable thermosensitive poly (organophosphazene) (PPZ) hydrogel with docetaxel (DTX-gel) to develop an effective therapeutic agent for patient with PC. Three days after inoculation of highly metastatic 44As3Luc cells into peritoneal cavity, the mice were intravenously or intraperitoneally administered with docetaxel alone (DTX-sol IV or IP), and intraperitoneally injected with DTX-gel. The anti-tumor activity was monitored by bioluminescence live imaging system. Compared to DTX-sol IV or IP, the tumor growth was significantly reduced in the DTX-gel treated mice (*p*<0.0001, *p*=0.0001). Furthermore, the survival rate was significantly increased in the DTX-gel treated mice compared to DTX-sol IV or IP treated mice (*p*<0.0001, *p*=0.0068). Our results demonstrated that DTX-gel suppresses peritoneal metastasis by continuing release of chemotherapy agent, which leads to increase the survival rate in a PC model. Therefore, biodegradable thermosensitive hydrogel with docetaxel system can be a good anti-cancer agent for PC.

## INTRODUCTION

Gastric cancer (GC) is the fourth most common cancer and the second leading cause of cancer-related death around the world [1]. One of important metastatic dissemination of GC is mainly occurred through spread of cancer cells to the peritoneal cavity, which is called peritoneal carcinomatosis (PC). Despite recent advances in treatments, the median survival of GC patients with PC is 3-6 months, and the survival rate is less than 1% [2, 3]. Therefore, to reduce mortality and to provide symptom relief for GC patients with PC, development of novel effective treatments are necessary.

GC patients with PC are usually treated with intravenously systemic chemotherapy without surgery because of poor survival even with chemotherapy. However, the systemic chemotherapy has a poor penetration of anti-cancer drugs into the peritoneal cavity due to the peritoneal-plasma barrier comprising of mesothelium and submesothelial tissues [4]. Therefore, to improve the penetration efficiency in the peritoneal cavity, intraperitoneal (IP) chemotherapy for patients with PC has been conducted, because IP chemotherapy methods can expose much higher drug concentration to peritoneal tumors than systemic chemotherapy [5, 6]. Previously, de Castro et al. conducted a randomized phase III trial, which demonstrated that administration of intraperitoneal anti-cancer drug in ovarian cancer patients with peritoneal dissemination had longer overall survival due to high local concentration of anti-cancer drug in peritoneal cavity [7]. Nonetheless, intraperitoneal administration has several limitations such as systemic and local toxicity including abdominal pain, adhesion formation, and chemical peritonitis caused by failure of control release of anti-cancer drugs [8].

For the IP chemotherapy, docetaxel has been investigated due to a high sensitivity against various malignancies compared to the other anti-cancer drugs such as paclitaxel, cisplatin and 5-FU. However, docetaxel is rapidly absorbed by peritoneal capillaries, followed by elimination via hepatic metabolism less than a day. Short retention time in the peritoneal cavity requires frequent injection of the anti-cancer drugs.

To improve retention time of anti-cancer drugs in the peritoneal cavity, various nanoparticulate-based delivery systems such as micelles, microspheres, and liposomes have been investigated [9–15]. Biodegradable thermosensitive hydrogels are ideal injectable biomaterials for medical applications including cancer therapy. The hydrogels are easy to mix with chemotherapy drugs and/or therapeutic peptides, and it could reversibly shift sol-gel status depending on temperature [16]. In addition, local injection of hydrogel with anti-cancer drug in tumor site can maintain the therapeutic effect in tumor tissue with high concentration and prolonged retention time [17–20]. Previously, we have designed intratumorally injectable polyphosphazene (PPZ) hydrogel for anti-cancer drug delivery system. The PPZ hydrogel were reversibly transit sol-gel status depending on temperature. Moreover, it was locally injectable with high concentration of anti-cancer drug at a limited tumor site with extended retention time [21].

In the present study, we have highlighted efficacy of the PPZ hydrogel application in terms of reducing systemic side effect of IP administration and extension the retention time of chemotherapeutic drugs in the local peritoneal cavity without systemic toxicity. To this end, we have established *in vivo* mouse PC models and treated PPZ hydrogel with anti-cancer drug. We have evaluated treatment efficiency of chemotherapy agent mixed PPZ hydrogel via tumor growth rate and mice survival time as well as confirmed bio-safety of the hydrogel through anatomical and histological examination.

## RESULTS

### Thermosensitive biodegradable hydrogel is a safe biocompatible polymer material

**Figure 1:**
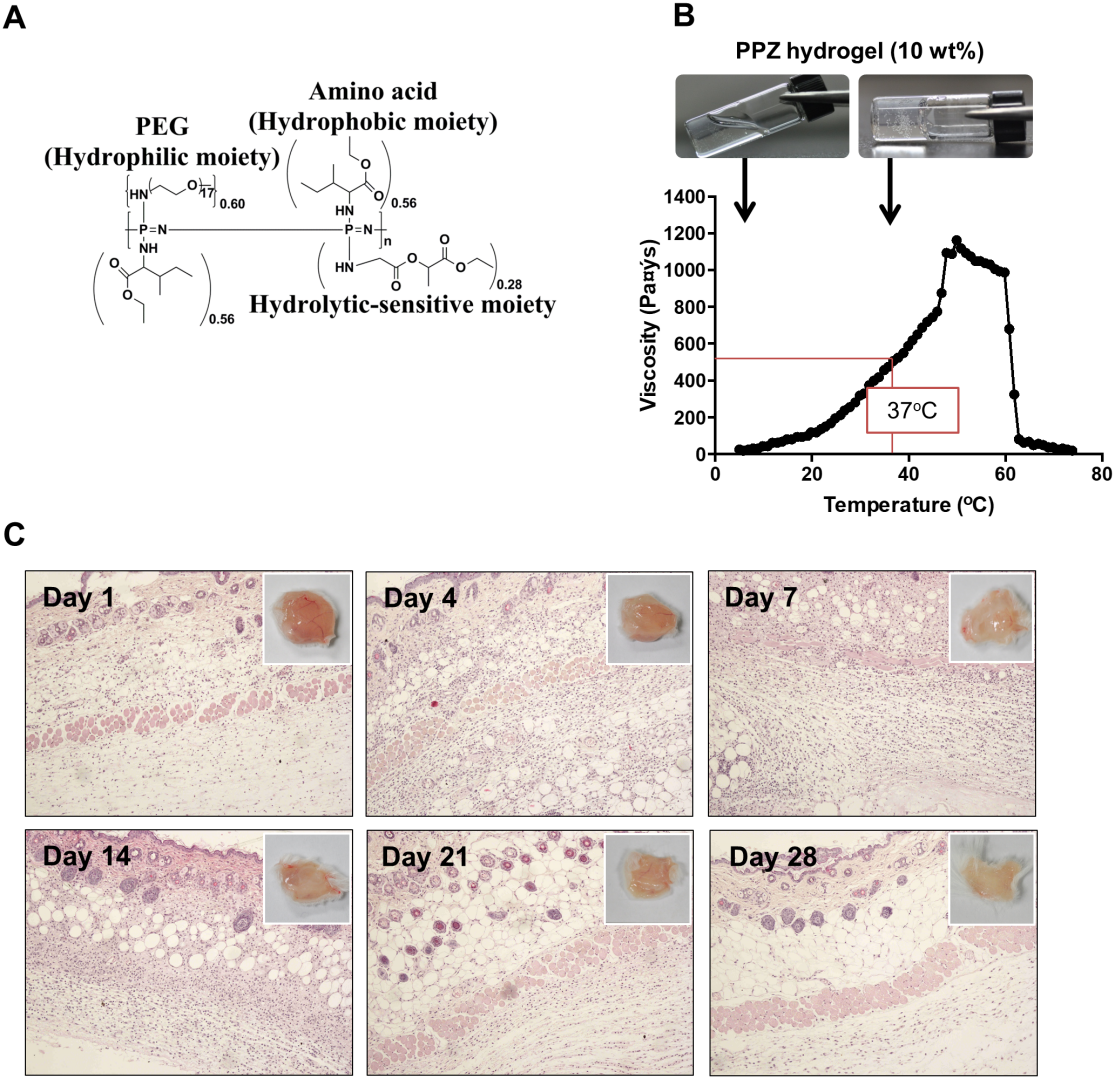
Properties of PPZ hydrogel and biocompatibility test **(A)** Structure of PPZ hydrogel. **(B)** Temperature dependent solution to gel transition with viscosity change of 10 wt % hydrogel. **(C)** Histologic examination of skin injection site during 28 days after inoculation of PPZ hydrogel. The upper right corner figures show the macroscopic images of skin on each day.

The molecular structure of biodegradable thermosensitive PPZ hydrogel is shown in Figure 1A. The characteristic ^1^H NMR peaks and GPC (Supplementary Figure 1A and 1B) were analyzed. The number average molecular weight (*M*_*n*_) and polydispersity index (PDI) of the PPZ hydrogel were 16161 g/mol and 1.919880, respectively (Supplementary Figure 1B). To understand the phase transition of hydrogel by temperature change, the variation of viscosity was monitored. The hydrogel was easily solved in PBS, which displayed solution-to-gel and gel-to-solution transition according to temperature change. The hydrogel was an injectable solution-type at 4°C (*T*_0_=5°C), while it was turned to a gel-type at body temperature (*V*37°C =506.25 Pa.s) (Figure 1B).

Prior to application of the hydrogel in pre-clinical animal experiments, we confirmed the safety and fitness of PPZ hydrogel in mouse body. The PPZ hydrogel was subcutaneously injected, and then the mice were sacrificed to analyze the acute immune response on day 1, 4, 7, 14, 21 and 28. Histological observation of each mouse skin showed that lymphocytes and macrophage were recruited around injection site until 14 days after injection (Figure 1C). However, there were no tissue damages or necrosis symptoms until 28 days. These results indicate that the biodegradable thermosensitive hydrogel is a safe injectable material for preclinical purpose.

### Intraperitoneal administration of hydrogel with docetaxel significantly suppresses peritoneal carcinomatosis (PC) growth in mouse animal model

First, we asked whether the 44As3Luc cells are conveniently detectable using radiance. The intensity of luciferase was positively correlated with the cells number in luciferase assay (Supplementary Figure 2A). In chemosensitivity assay, the 44As3Luc cell line revealed moderate sensitivity against to docetaxel with IC50 below 1 μg/mL at 96h (Supplementary Figure 2B).

**Figure 2:**
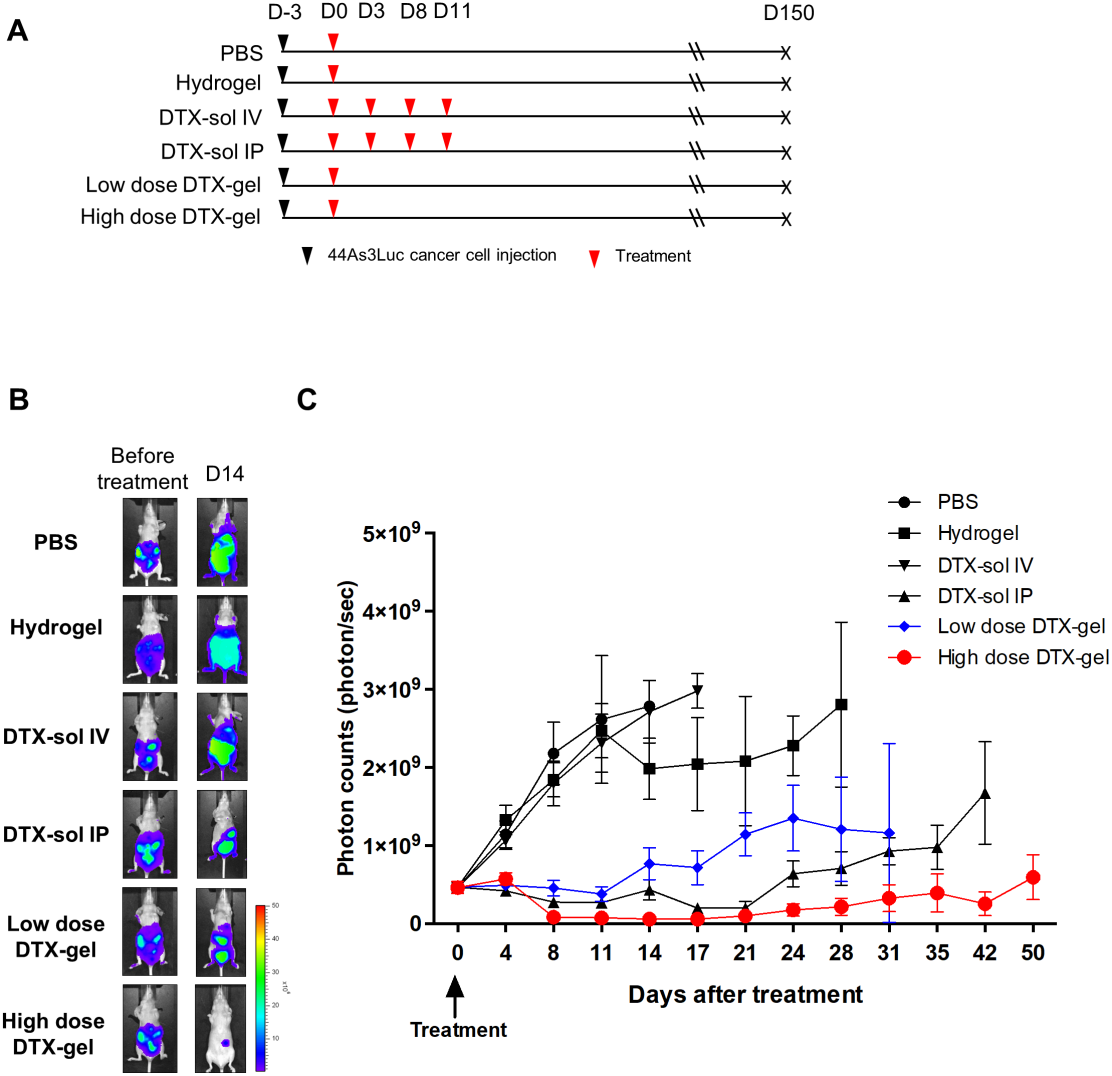
Analysis of *in vivo* bioluminescence imaging in peritoneal carcinomatosis (PC) mouse model The bioluminescence images were obtained until 50 days after treatment. **(A)** Schedule of cell injection and PBS, hydrogel, DTX-sol IV or IP, and low dose or high dose DTX-gel treatment. **(B)** Comparison of bioluminescence imaging between each group. **(C)** Analysis of photon counts from bioluminescence images.

Next, we analyzed an anti-cancer drug effect of the hydrogel with docetaxel for peritoneal dissemination of GC. The *in vivo* mouse PC models were established via inoculation of 44As3Luc cancer cells into the peritoneal cavity of nude mice, and the mice were treated with six kinds of reagent (Figure 2A). The therapeutic efficacy was assessed by measuring the intensity of bioluminescence signals (Figure 2B and 2C, Supplementary Figure 3C-3F). The photon counts were significantly diminished in low dose DTX-gel and high dose DTX-gel groups compared to both PBS and hydrogel control groups (*p*<0.001). In comparison between DTX-gel groups and DTX-sol groups, DTX-gel groups (both low and high doses) revealed significantly low photon counts compared to DTX-sol IV group. Notably, single treatment of high dose DTX-gel group showed lower photon counts than 4 times treatment of DTX-sol IP group (*p*=0.0001). Collectively, these data demonstrate that treatment of DTX with the hydrogel strongly and continuously suppresses PC growth.

### Anatomic and histologic evaluation of PC

**Figure 3:**
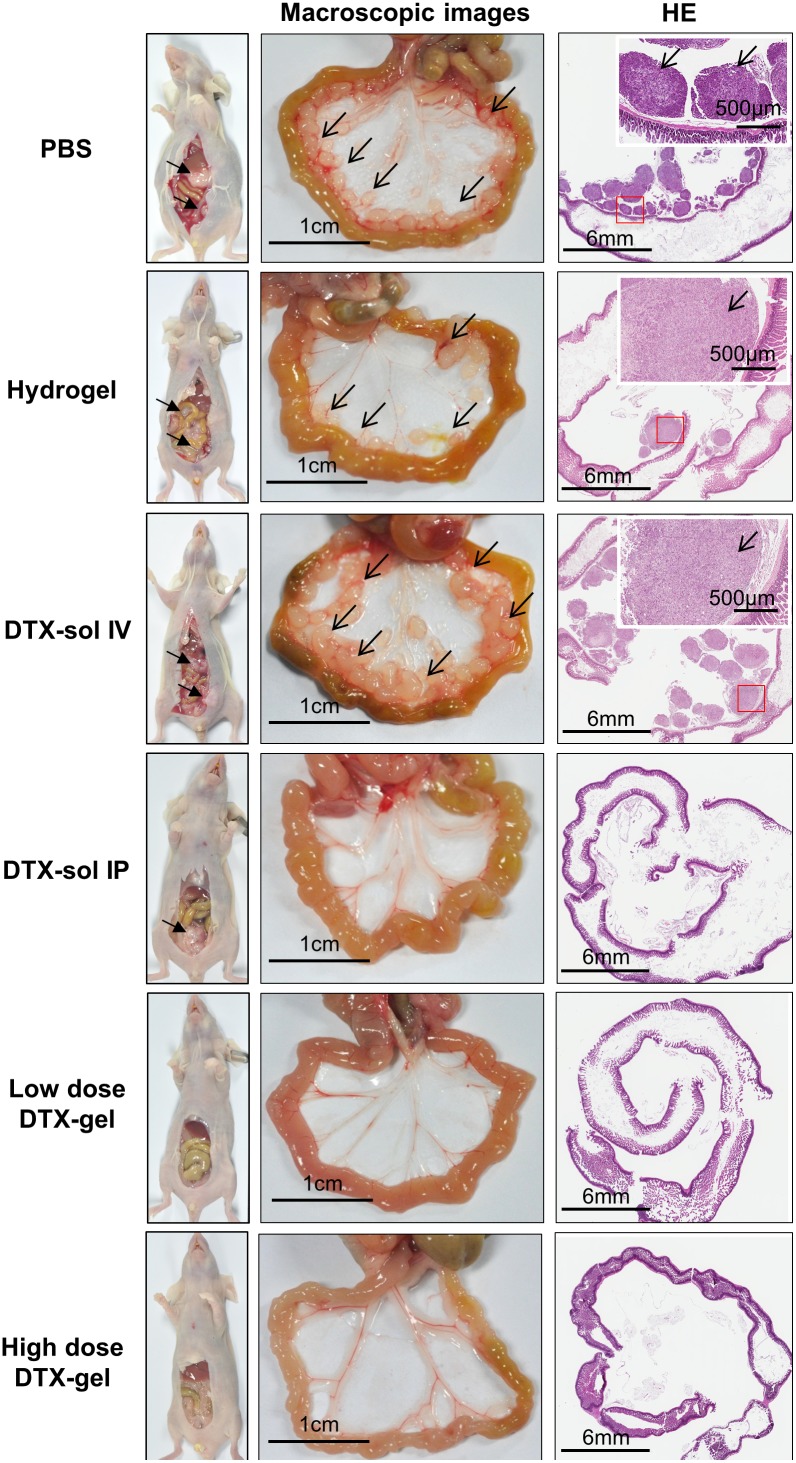
Macroscopic and histologic feature of mesentery tumor nodules On 14 days after treatment, one mouse from each group was randomly selected and sacrificed. Middle of figures indicate the macroscopic images of mesentery in each group. Black bar indicates 1 cm scale and black arrow indicates mesentery tumor nodules. Right side of figures indicate hematoxylin and eosin staining of intestine and mesentery tumor nodules. Black bars of left bottom and right upper site indicate 6 mm and 500 μm, respectively.

To further elucidate the tumor suppressive effect of DTX-gel treatment, we confirmed anatomical and histological morphology of the peritoneal tumors in each mouse group (Figure 3). The peritoneal metastasized tumors were observed in PBS, hydrogel, and DTX-sol IV groups, whereas low dose DTX-gel and high dose DTX-gel groups did not induce the peritoneal metastasized tumors. More detail, mesentery nodules were observed in PBS and DTX-sol IV groups on day 8 and 14. In hydrogel group, ascites and mesentery nodules were found on day 8, 14 and 28 (Figure 3 and Supplementary Figure 4). However, we could not observe ascites and mesentery nodules in treated mice with low dose DTX-gel and high dose DTX-gel groups. Moreover, the presence of metastasized tumors in stomach, kidney, spleen, liver, and mesentery were histologically confirmed in all mouse groups except high dose DTX-gel group (Supplementary Figure 5A-5D). The tumor area and numbers were distinctly increased in PBS, hydrogel, DTX-sol IV, and DTX-sol IP groups, while both tumor area and numbers were reduced in high dose DTX-gel group (Supplementary Figure 6A and 6B; Table 1).

**Table 1:** Tumor area (mm2) of each mouse

Group	Day 8 (mm^2^)	Day 14 (mm^2^)	Day 28 (mm^2^)
PBS	72.3765	96.3992	
Hydrogel	73.9286	170.9322	365.0136
DTX-sol IV	80.8297	193.1357	
DTX-sol IP	10.3247	55.3224	294.4449
Low dose DTX-gel	23.9267	47.4284	210.9437
High dose DTX-gel	0.8146	2.1939	20.7473

Additionally, to quantitatively evaluate the tumor cell growth in peritoneal cavity, we analyzed copy number of human carcinoembryonic antigen (CEA) from peritoneal wash samples on 8, 14 and 28 days. The CEA level was increased in PBS, hydrogel and DTX-sol IV groups, while the CEA level was decreased in DTX-sol IP and high dose DTX-gel groups on 8, 14 and 28 days (Supplementary Figure 7A and 7B). Taken together, these results demonstrate the tumor suppressive effect of hydrogel with high dose docetaxel treatment in development of peritoneal metastasized GC tumors.

### Treatment of docetaxel with hydrogel prolonged survival of mice with PC

**Figure 4:**
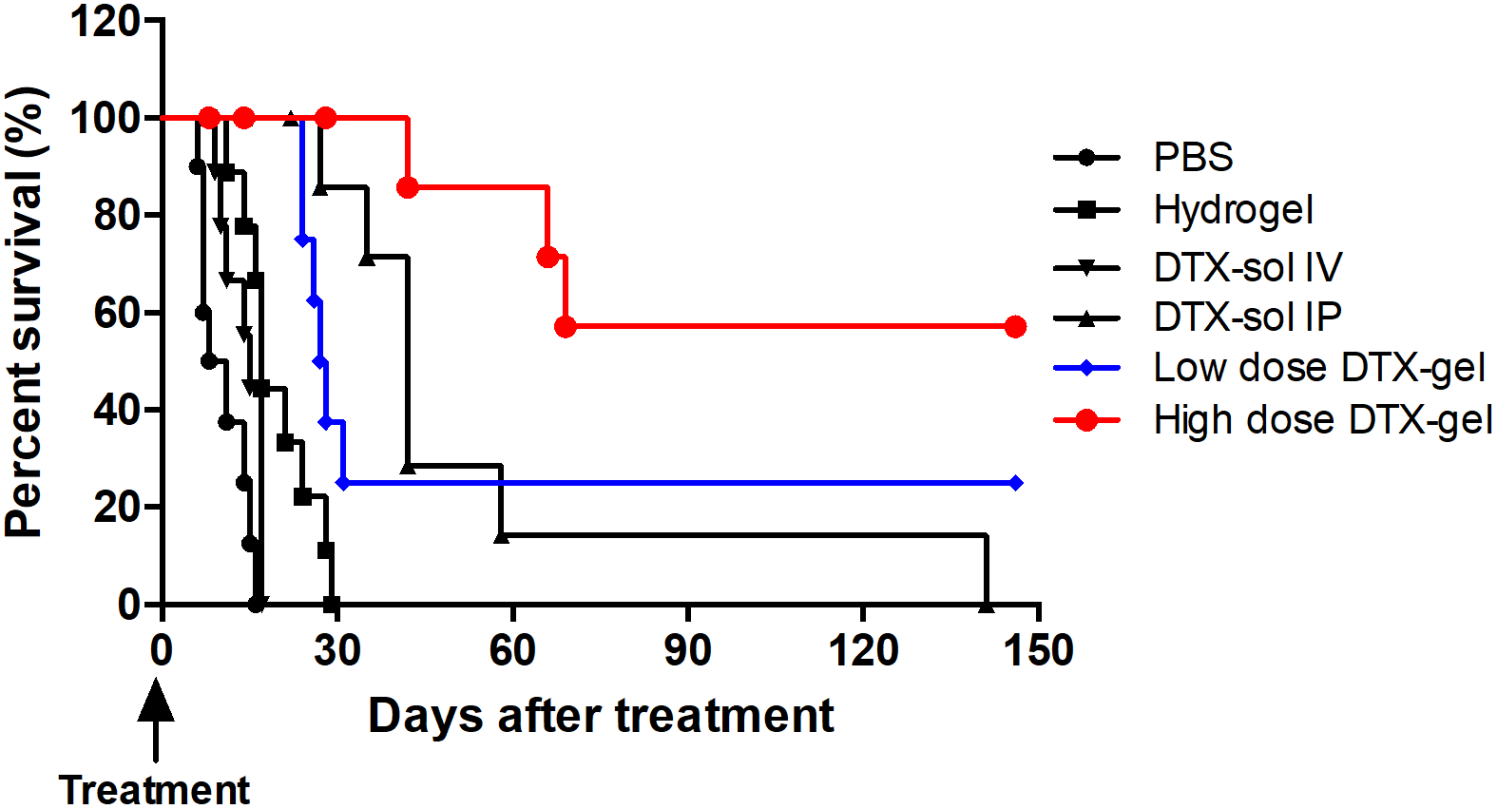
Proportion of survival Kaplan-Meier analysis of survival rates of each group until 150 days.

Finally, to further evaluate an efficacy of treatment of hydrogel with docetaxel in mouse with PC survival, we performed Kaplan-Meier analysis. For 150 days after treatment, the median survival time was 9.5, 17, 15, 42, 27.5 and 102 days in the PBS, hydrogel, DTX-sol IV, DTX-sol IP, low dose DTX-gel, and high dose DTX-gel, respectively (Figure 4). The mice treated with low dose DTX-gel and high does DTX-gel had significantly better survival than those with other treated mice groups (*p*<0.0001). In addition, the mice of high dose DTX-gel group revealed significantly more prolonged survival time compared to mice of DTX-sol IP group (*p*=0.0068). At the end of this study (150 days after treatment), 5 mice were still alive (2 mice in the low dose DTX-gel group and 3 mice in high dose DTX-gel group). Taken together, these data are of significance, as they indicate that treatment of high dose docetaxel with hydrogel safely and effectively suppresses PC growth.

## DISCUSSION

**Figure 5:**
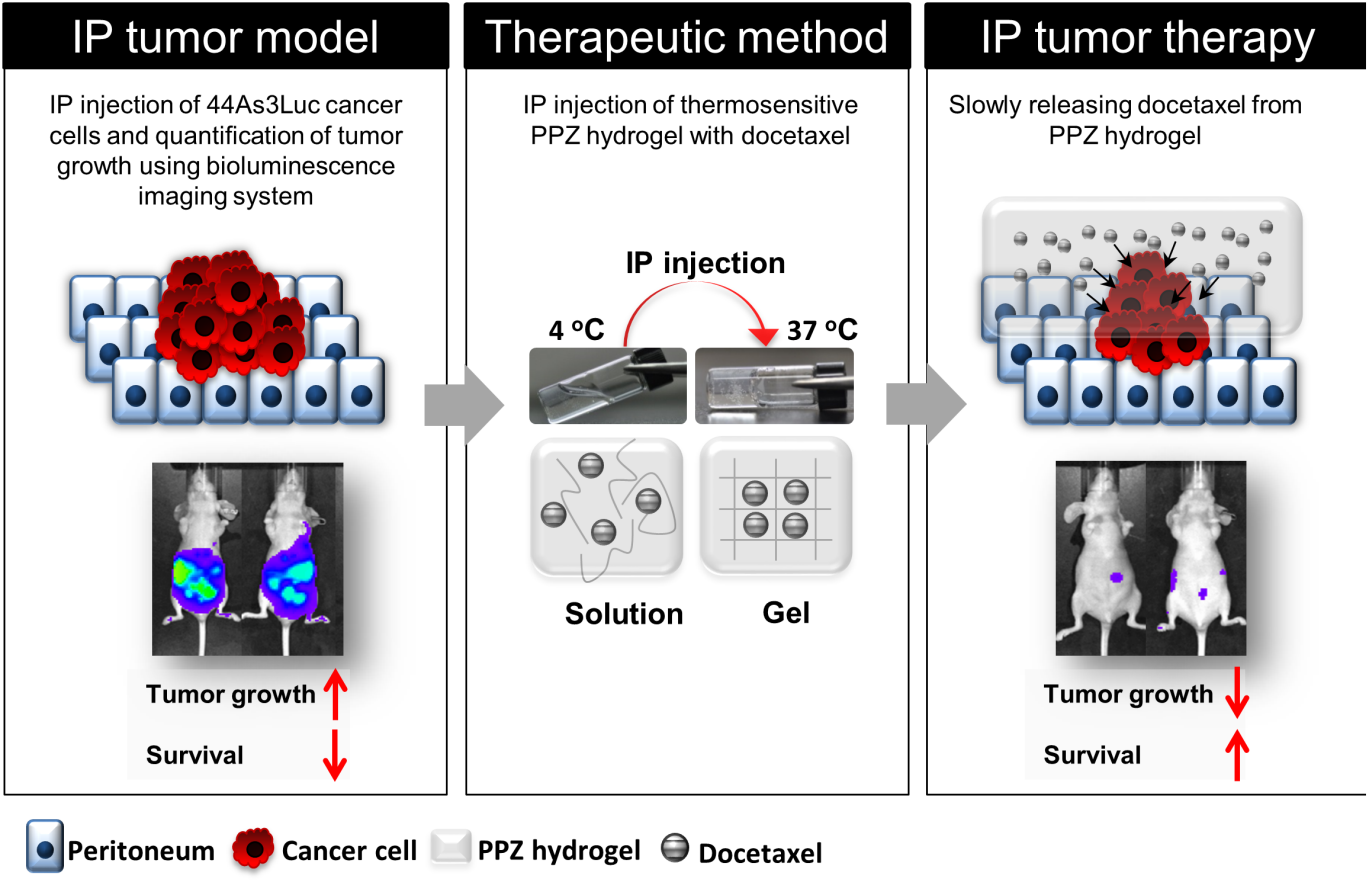
Peritoneal carcinomatosis therapy model using injectable thermosensitive biodegradable hydrogel with docetaxel Tumor growth is quantitatively analyzed using bioluminescence imaging system. Hydrogel with docetaxel system can slowly release the anti-cancer drug in body, therefore, long-term peritoneal carcinomatosis therapy is possible without severe toxicity.

PC is a major cause of death in GC patients, and it is known to be an intractable problem. Although intravenous chemotherapy has been known to be the most effective standard treatment for peritoneal disease, it frequently results in bone marrow suppression and other complications due to systemic toxicity. Previously, many research groups have developed several peritoneal disease treatment methods, including systemic chemotherapy, intraperitoneal chemotherapy, peritonectomy, and thermotherapy [28–31]. Nonetheless, they did not gain much achievements due to potential limitation of repeated intraperitoneal administration of chemotherapeutic agent and complications. To address this issue, we applied biodegradable thermosensitive PPZ hydrogel system as a novel treatment approaches, which can deliver continuously high concentration of chemotherapeutic agent locally but with less systemic toxicity or complications (Figure 5).

A critical requirement of biomaterials used in living bodies is biocompatibility. Generally, degradation of synthetic polymers occur by hydrolysis of their ester linkages and/or by enzymatic reactions. In this regard, depolymerized gel materials are possible to provoke both local and systemic inflammatory responses. To ensure bio-safety of our gel, we evaluated acute toxicity of the hydrogel via subcutaneous injection of the gel on wild-type Balb/c mice. Although a few lymphocytes and macrophages were recruited, almost no necrosis and no severe inflammation were observed at the local gel injected site. In systemic toxicity evaluation analysis, morphological changes or immune responses were not found in heart, lung, intestine, and kidney tissues on 8, 14 and 28 days after our PPZ hydrogel injection. In liver tissues, foamy macrophages were observed in hydrogel, low dose DTX-gel, and high dose DTX-gel groups, however it was a negligible immune response. In addition, there were no significant changes of body weight in all mice until 21 days (Supplementary Figure 8). Taken together, these results reveal that our PPZ hydrogel is not only locally, but also systemically biocompatible for clinical purpose of applications.

Another novel aspect of this study was that we successfully demonstrated functional relevance of our biodegradable thermosensitive PPZ hydrogel in PC therapy. Despite the fact that several cycles treatment of the docetaxel into peritoneal cavity raised toxicity problems, few previous studies emphasized significant increase of patients survival in ovarian cancer and GC with PC [7, 32]. On the contrary, we performed treatment of the docetaxel in DTX-sol IP group, as similar as possible clinical trials. The docetaxel solution was injected by 4 times with 2 mg/kg dosage. Since the most attractive advantage of PPZ hydrogel is continuous time dependent release of anticancer drug, we conducted one time treatment of the gel into mice peritoneal cavity of low dose DTX-gel and high dose DTX-gel groups. We noted that anti-cancer effect was more improved in DTX-sol IP group than DTX-sol IV treated mice. Furthermore, we found that intraperitoneal administration of PPZ hydrogel with docetaxel had better anti-cancer effect than DTX-sol IV and DTX-sol IP. More interestingly, the initial treatment response of DTX-sol IP group was comparable with high dose DTX-gel group in the bioluminescence images. However, this phenomenon was not sustained for long time (less than 4-week). Although CEA level was similar between DTX-sol IP group and high dose DTX-gel group, it may be due to analysis of only floating cancer cells obtained from washing cytology, except attached cancer cell, in peritoneal cavity. Nonetheless, we clearly showed that hydrogel with high dose docetaxel group suppressed both attached and floating cancer cells, and these results highlight that PPZ hydrogel is able to slowly release high concentration of docetaxel for long time period in the peritoneal cavity without systemic toxicity.

Previously, we reported that thermosensitive hydrogel with paclitaxel system using HSC44Luc cell line can be used to treat carcinomatosis mouse model with minimal side effects and prolong the retention of therapeutic effect [33]. However, the study had some limitations to evaluate the PC therapy because only five mice were used for each group. In the current report, we have established a PC mouse model using ten mice of each group, high metastatic GC cell line (44As3Luc) and *in vivo* bioluminescence imaging system. Furthermore, our study used docetaxel as a therapeutic agent for PC instead of paclitaxel, because docetaxel has a broad spectrum of antitumor activity against various malignancies breast, gastric and ovarian cancers, and leads to disruption of microtubule mediated cellular function in the G2 to M transition, and cell death [34–36]. Moreover, phase II clinical study reported that docetaxel was active in paclitaxel-resistant ovarian and peritoneal dissemination patients [37]. We thus applied the docetaxel and PPZ hydrogel to validate synergistic effect for PC treatment with safety and effectiveness.

In conclusion, intraperitoneal administration of hydrogel with docetaxel system demonstrated a significant therapeutic effect against PC mice model of GC. The tumor growth was more suppressed in high dose DTX-gel group than DTX-sol IP group after treatment, and DTX-gel systems led to longer median survival time. Therefore, this study suggests that PPZ hydrogel with anti-cancer drug system can be an effective treatment and low toxicity for patients with PC and can be considered as a new strategy for PC therapy.

## MATERIALS AND METHODS

### Preparation of biodegradable thermosensitive hydrogels

The biodegradable thermosensitive hydrogel was synthesized by similar procedures as previous reports [22] and characterized by multinuclear nuclear magnetic resonance (NMR) spectroscopy, gel permeation chromatography (GPC), and rheometry analysis. The hydrogel, poly (dichlorophosphazene), contained IleOEt HCl, AMPEG 750 and GlyLacOEt OA. Briefly, to prepare 10 wt% aqueous hydrogel solution, the synthesized hydrogel was dissolved in phosphate buffered saline (PBS) with stirring at 4°C for 3 days. When the hydrogel was completely dissolved in PBS, the 10 wt% hydrogel was physically mixed with docetaxel by stirring at 4°C for 3 days.

### Biocompatibility test for PPZ hydrogel

The PPZ hydrogel was injected into subcutaneous in 6-week Balb/c mice (Orient Bio Inc., Seoul, Korea). The mice were randomly sacrificed on 1, 4, 7, 14, 21 and 28 days. For observation of tissue toxicity, skin and liver were immediately collected and fixed in 10% neutral-buffered formaldehyde solution, and embedded in paraffin. All samples were stained with hematoxylin and eosin.

### Luciferase activity and cytotoxicity assays in 44As3Luc human GC cell line

To establish *in vivo* mouse PC models, we selected a highly peritoneal metastatic GC cell line, 44As3Luc, which was established from an ascitic fluid of a peritoneal metastasis mouse models for human scirrhous stomach cancer [23, 24]. 44As3Luc cells were grown in RPMI1640 supplemented with 10% FBS and 1% penicillin and streptomycin. Cells were maintained at 37°C under an atmosphere of 5% CO_2._

44As3Luc cells were seeded in 96-well plate. After 24 hours, cells were harvested and luciferin (Applied Biosystems, Foster City, CA) was added to cell extracts. The luciferase activity was measured by luminometry using TR717 solution (Applied Biosystems) [25].

44As3Luc cells were plated in 96-well tissue culture plates at 5 × 10^3^ cells/well. After 24 hours incubation at 37°C, the cells were treated with docetaxel solutions. Cellular viability was assessed by CCK-8 (Dojindo, Kumamoto, Japan) [26, 27].

### Preclinical trials using balb/c-nu mice

44As3Luc cells (1 × 10^6^ cells per mouse) were intraperitoneally injected in six-week-old female athymic nude mice (Balb/c-nu; Orient Bio Inc., Seoul, Korea). The mice were randomly divided into six groups (The photon counts were no significantly differences between each group; Supplementary Figure 3A and 3B), and treated as follows: 1) intraperitoneal (IP) injection of PBS (PBS group, n=10), 2) IP injection of hydrogel without docetaxel (hydrogel group, n=10), 3) intravenous (IV) injection of 2 mg/kg × 4 docetaxel solution without hydrogel (DTX-sol IV group, 2 times/week × 2, n=10), 4) IP injection of 2 mg/kg × 4 docetaxel solution without hydrogel (DTX-sol IP group, 2 times/week × 2, n=10), 5) IP injection of PPZ hydrogel physically mixed with 2 mg/kg docetaxel (low dose DTX-gel) 6) IP injection of PPZ hydrogel physically mixed with 8 mg/kg docetaxel (high dose DTX-gel) (Table 2). From three days after cell injection, these treatments were administered. On the 8, 14 and 28 days after treatment, one mouse of each group was randomly selected and sacrificed for gross and microscopic examination of intraperitoneal organs and suspicious tumor nodules. This animal study was approved by the IACUC of Seoul National University Hospital.

**Table 2:** Treatment groups

Group	Docetaxel dose	Injection number	Injection site	Number of mice
PBS	∙	1	Intraperitoneal cavity	10
Hydrogel	∙	1	Intraperitoneal cavity	10
DTX-sol IV	8 mg/kg, 2 times/week × 2	4	Intravenous	10
DTX-sol IP	8 mg/kg, 2 times/week × 2	4	Intraperitoneal cavity	10
Low dose DTX-gel	2 mg/kg	1	Intraperitoneal cavity	10
High dose DTX-gel	8 mg/kg	1	Intraperitoneal cavity	10
Total				60

### *In vivo* bioluminescence imaging

Mice were anesthetized with isoflurane and intraperitoneally injected 150 mg/kg of D-luciferin (MIP, Molecular Imaging Products, Bend, Oregon, USA). Bioluminescence images were obtained with exposure time of 30 seconds using IVIS-100 system (Xenogen Corp., Alameda, CA). The luciferase activity was measured every twice a week.

### Histology

After sacrifice of mice, organs were immediately fixed in 10% neutral-buffered formaldehyde solution, and embedded in paraffin. The paraffin embedded sections were cut at 4 *μm* thickness and mounted on poly-L-lysine-coated slides. Sections were deparaffinized in xylene, and stained with hematoxylin and eosin. To calculate the tumor area, the Aperio Scan Scope CS (Leica Biosystems, Newcastle, UK) equipment was used for scanning all H&E slides and then, the tumor area from slide images was calculated by Aperio ImageScope (Leica) software. All samples were examined by expert pathologists (SJ Byeon and WH Kim).

### RNA isolation and human carcinoembryonic antigen (CEA) real-time RT-PCR

On 8, 14 and 28 days, 2 ml of saline buffer were injected into the peritoneal cavity. After that one milliliter of peritoneal wash samples was collected with a 21 G needle. After centrifugation at 3,000 rpm for 1 minute, RNA was isolated from the cell pellets by TRIzol Reagent (Invitrogen, Carlsbad, CA), and then cDNA was synthesized (Invitrogen). The cDNA was amplified by the forward primer 5′-gat cag ggg aaa atc tga acc-3′ and the reverse primer 5′-ggg tcc tgt tgt cat tgg ac-3′ for CEA standard curve. The forward primer 5′-TGG ATC CTA TAC GTG CCA AGC-3′ and the reverse primer 5′-GAT GAA GGG TTT GGG TGG CT-3′ were used to measure the CEA level from wash samples. Real-time PCR was performed under the following conditions: 5 minutes at 95°C; 40 cycles of 30 seconds at 95°C, 15 seconds at 57°C, and 30 seconds at 72°C; 2 minutes at 72°C.

### Statistical analysis

The student *t* test and two-way anova were used for comparison of photon count means among groups. Kaplan-Meier survival analysis with a log-rank test was used for comparison of survival rate of each group. *p*< 0.05 was considered to be statistically significant. Data analysis was performed using Graphpad Prism version 5.0 (GraphPad Software, San Diego, CA).

## SUPPLEMENTARY MATERIALS FIGURES



## Abbreviations

GC: gastric cancer
qRT-PCR: quantitative real-time polymerase chain reaction
CEA: carcinoembryonic antigen
IP: intraperitoneal
IV: intravenous
DTX: docetaxel
DTX-gel: hydrogel with docetaxel
