# Supplementary material for: Improvement of anti-cancer drug efficacy via thermosensitive hydrogel in peritoneal carcinomatosis in gastric cancer
Source: Oncotarget. 2017 Nov 6;8(65):108848–58. doi: 10.18632/oncotarget.22312 (PMC5752486; doi:10.18632/oncotarget.22312)
Supplement: Supplementary file 1 [file oncotarget-08-108848-s001.pdf]

Improvement of anti-cancer drug efficacy via thermosensitive hydrogel in peritoneal carcinomatosis in gastric cancer

SUPPLEMENTARY MATERIALS

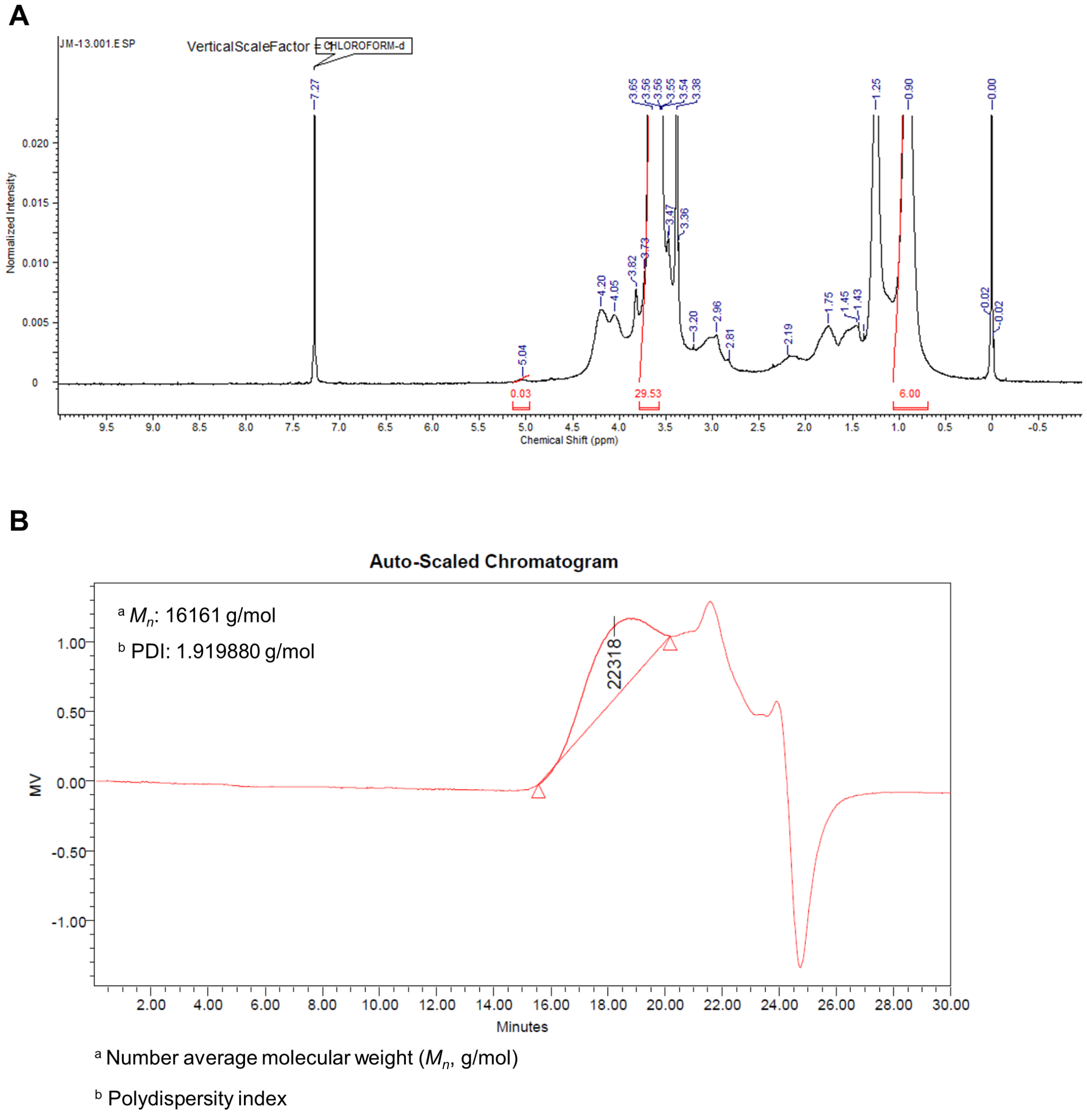

Supplementary Figure 1: Characteristics of synthesized PPZ hydrogel. (A) ^{1}H multinuclear nuclear magnetic resonance (NMR) spectrum of PPZ hydrogel. **(B)** gel permeation chromatography (GPC) analysis of PPZ hydrogel. [a] Number average molecular weight (M_n, g/mol). [b] Polydispersity index.

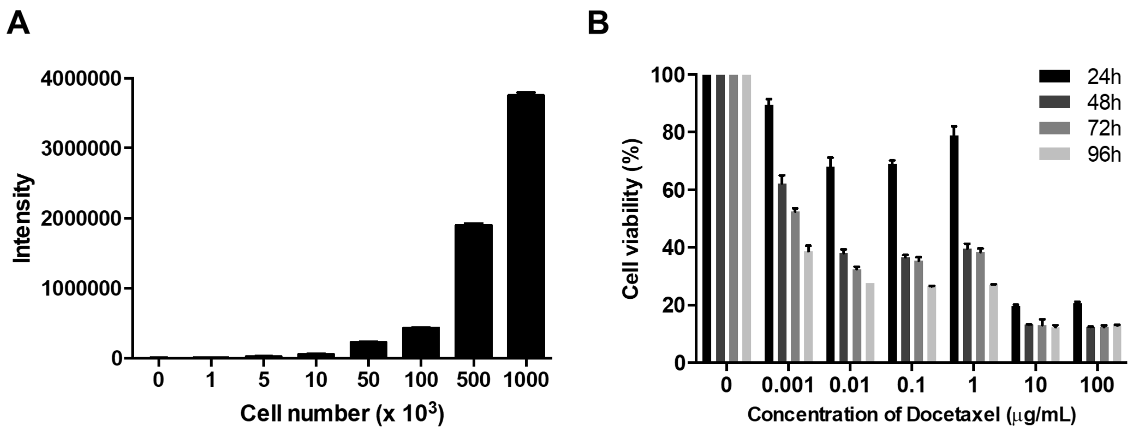

Supplementary Figure 2: *In vitro* study using high metastatic 44As3Luc cancer cell line. **(A)** Luciferase activity of 44As3Luc cancer cells depending on cell number. **(B)** 44As3Luc cancer cell viability against docetaxel.

A. Before treatment

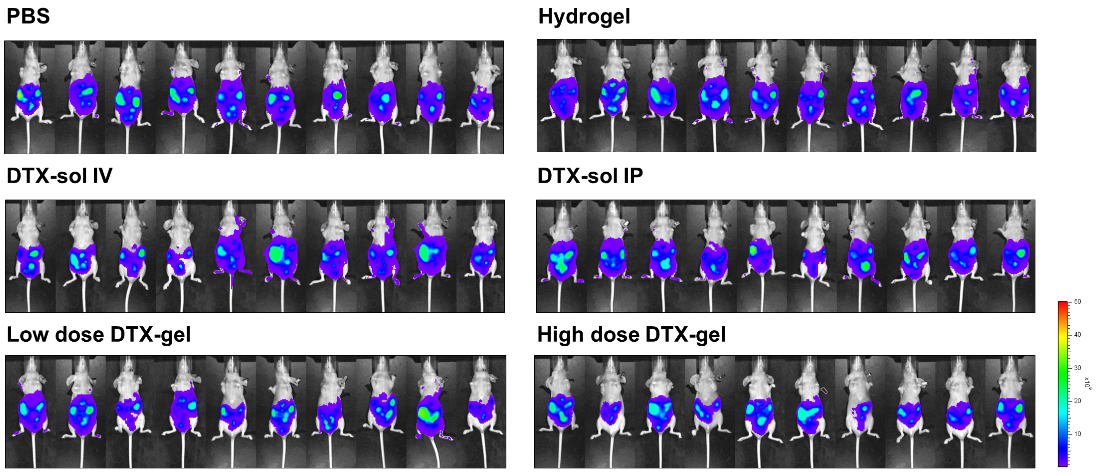

B. Photon counts (before treatment)

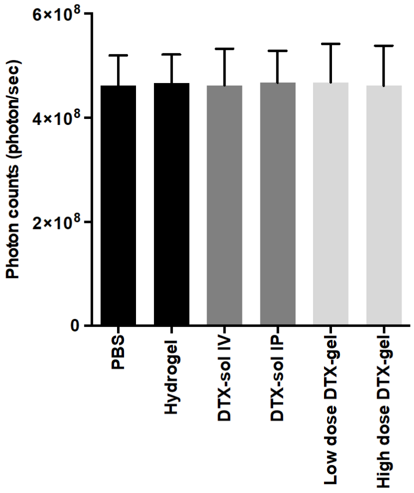

(*Continued*)

C. Day 8

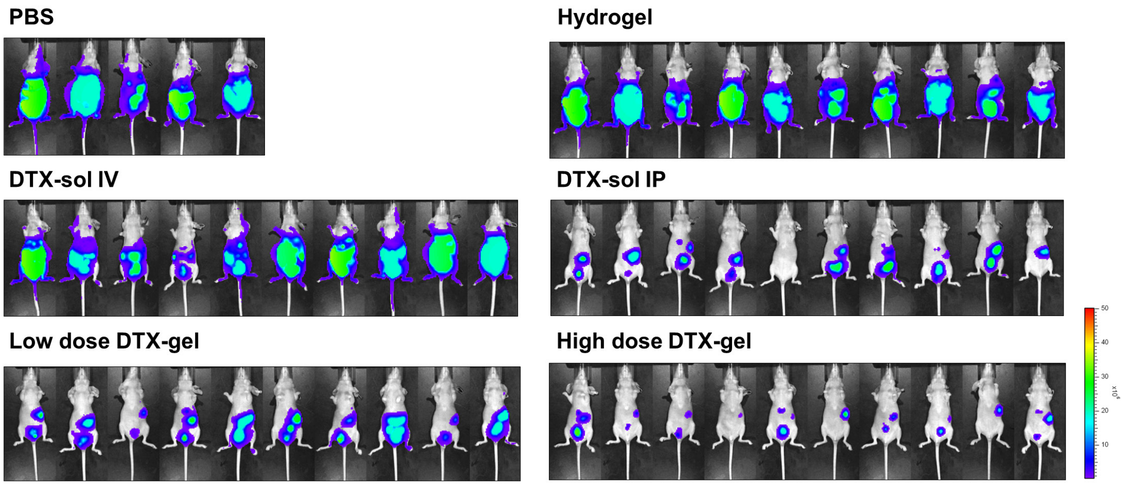

D. Day 14

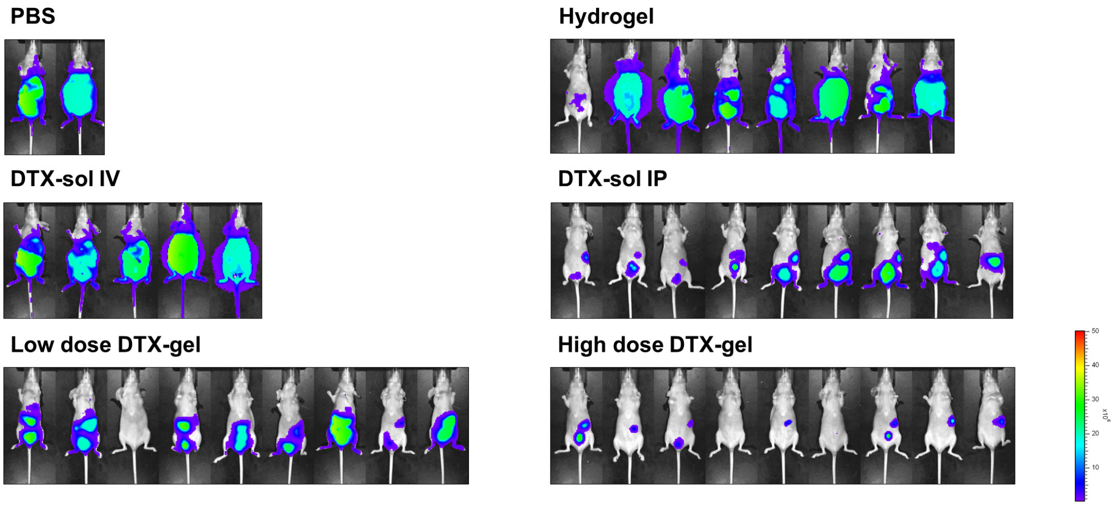

(*Continued*)

E. Day 28

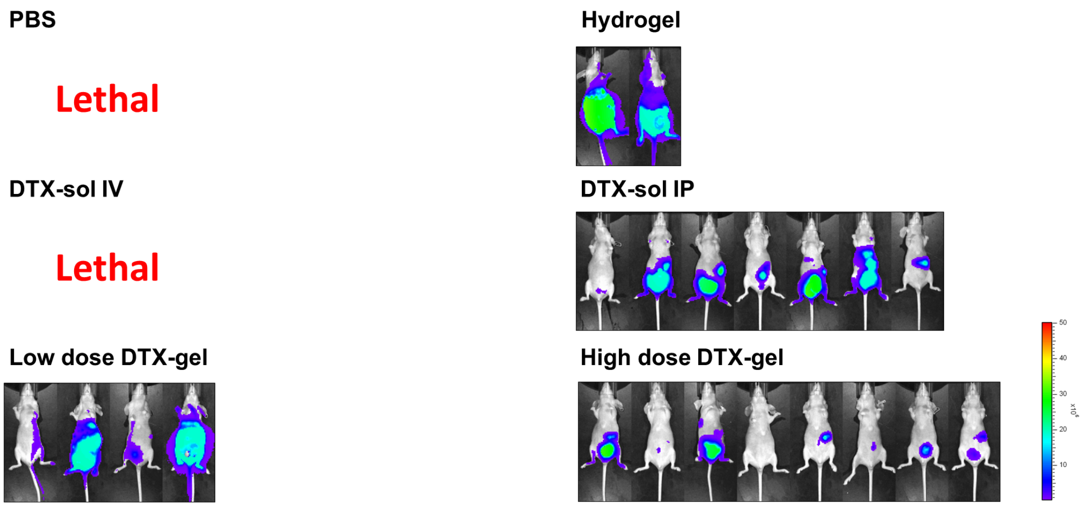

F. Day 50

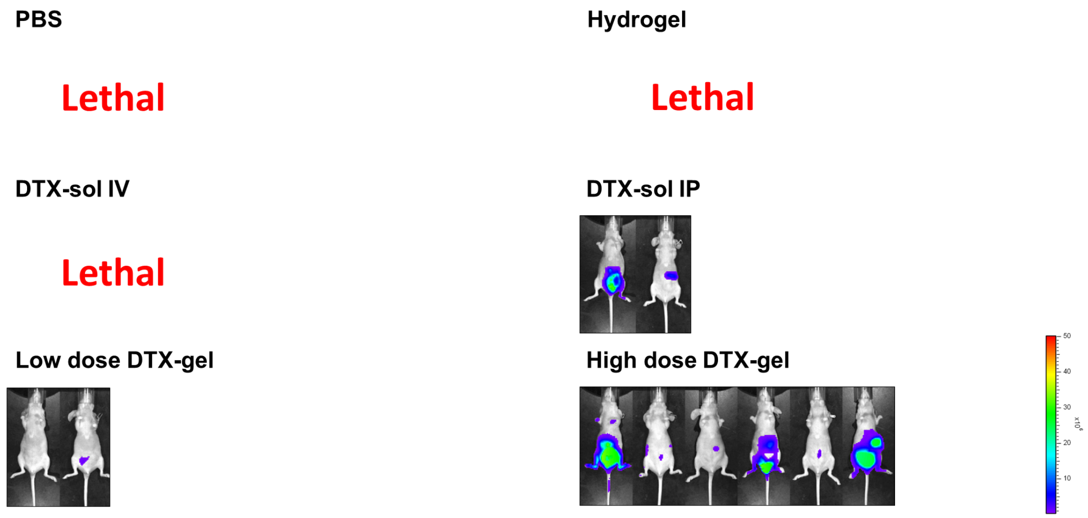

Supplementary Figure 3: Grouping of each mouse using bioluminescence imaging system. (A) *In vivo* bioluminescence imaging of each group. **(B)** Photon counts were measured on day 3 after 44As3Luc cell inoculation. Ten mice were assigned in each group. The average of photon counts was similar between each group and it was no significant difference each group. **(C-F)** Bioluminescence imaging of each group until 50 days after treatment.

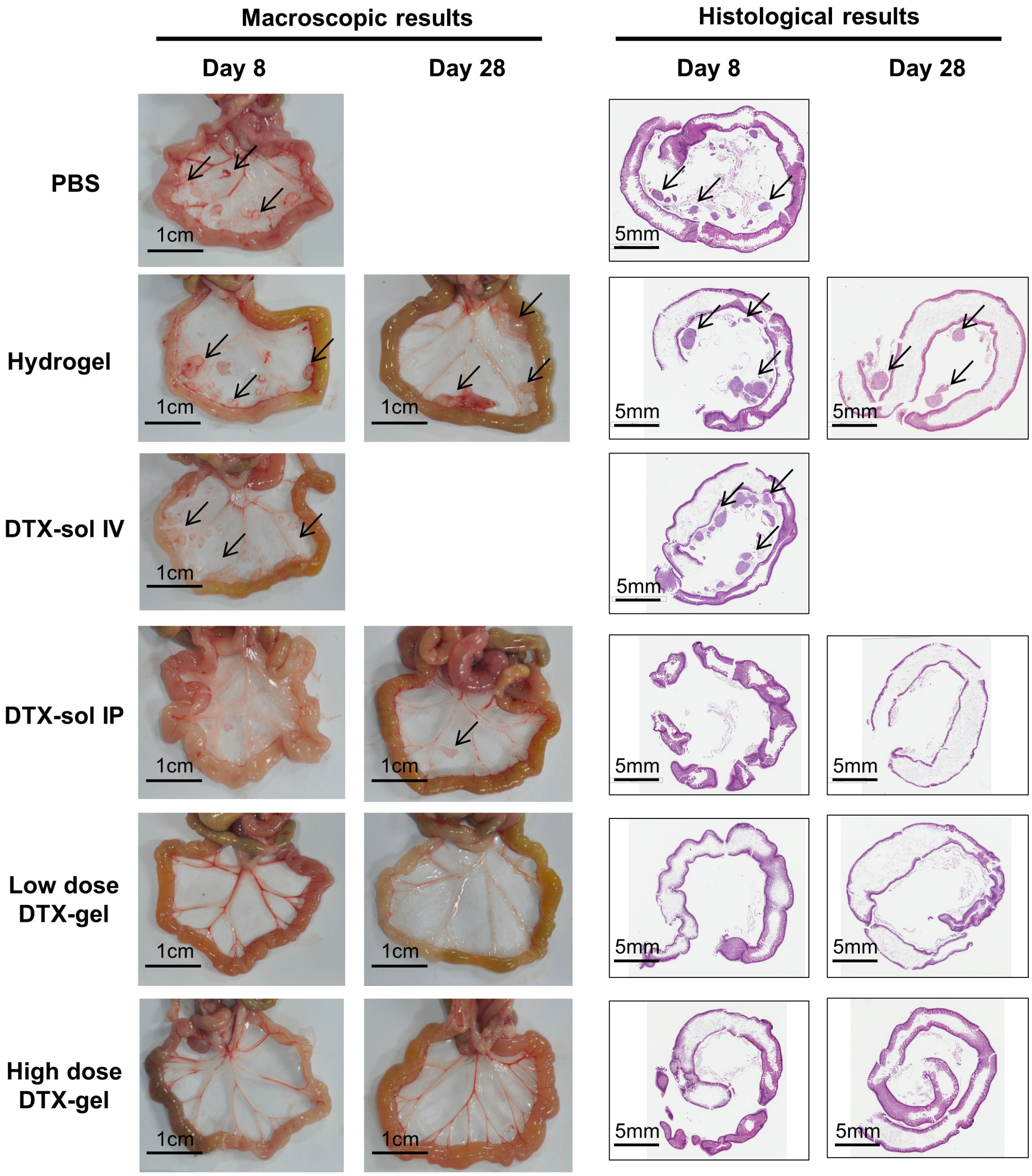

Supplementary Figure 4: Macroscopic appearance of mesentery tumors. Macroscopic and microscopic observation of mesentery tumor nodules on 8 and 28 days after treatment. Black arrows indicate mesentery tumor nodules.

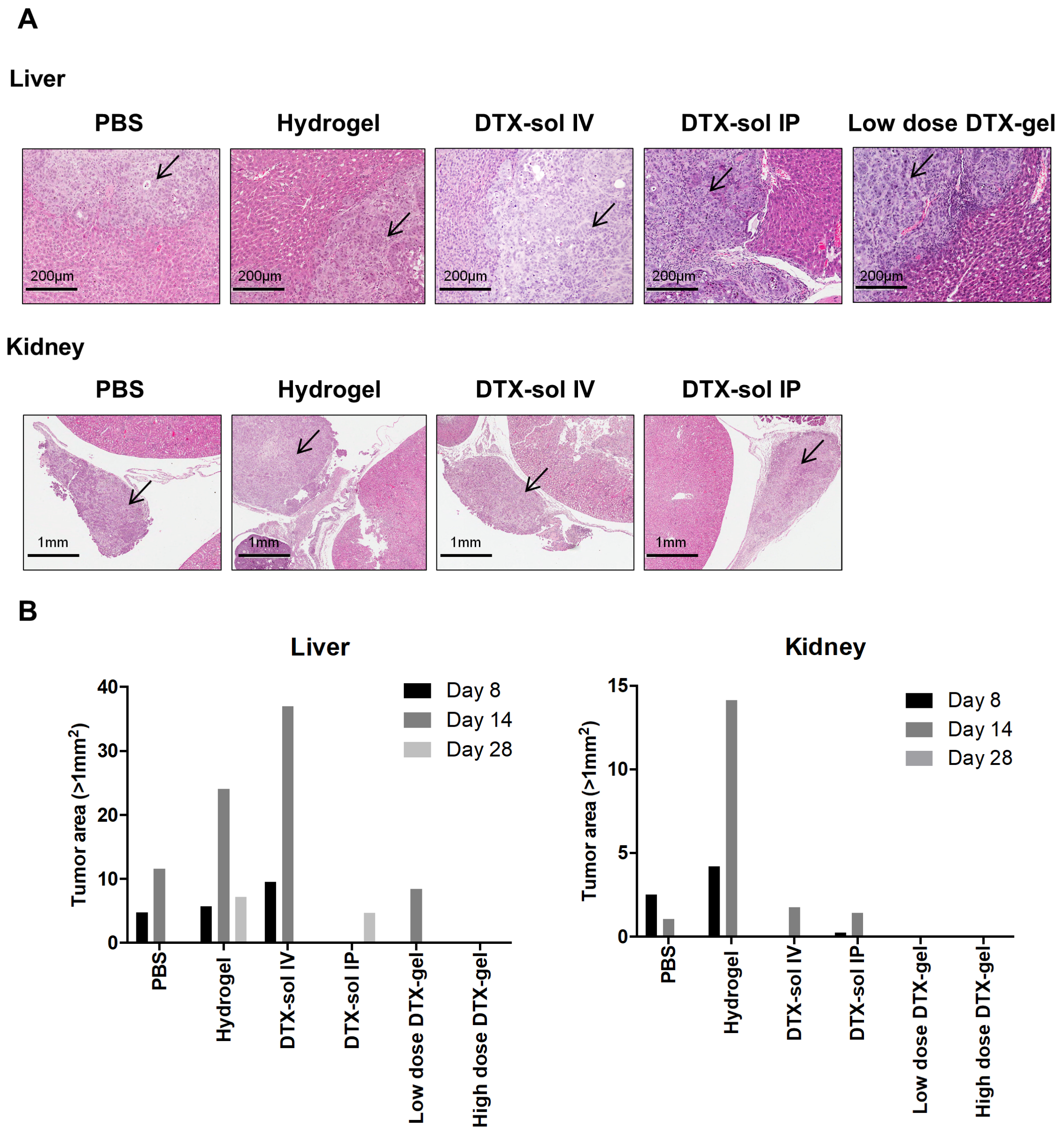
A
Liver
PBS
Hydrogel
DTX-sol IV
DTX-sol IP
Low dose DTX-gel
200μm
Kidney
PBS
Hydrogel
DTX-sol IV
DTX-sol IP
1mm
B
Liver
Kidney
Tumor area (>1mm²)
Day 8
Day 14
Day 28
0
10
20
30
40
5
15
PBS
Hydrogel
DTX-sol IV
DTX-sol IP
Low dose DTX-gel
High dose DTX-gel

(*Continued*)

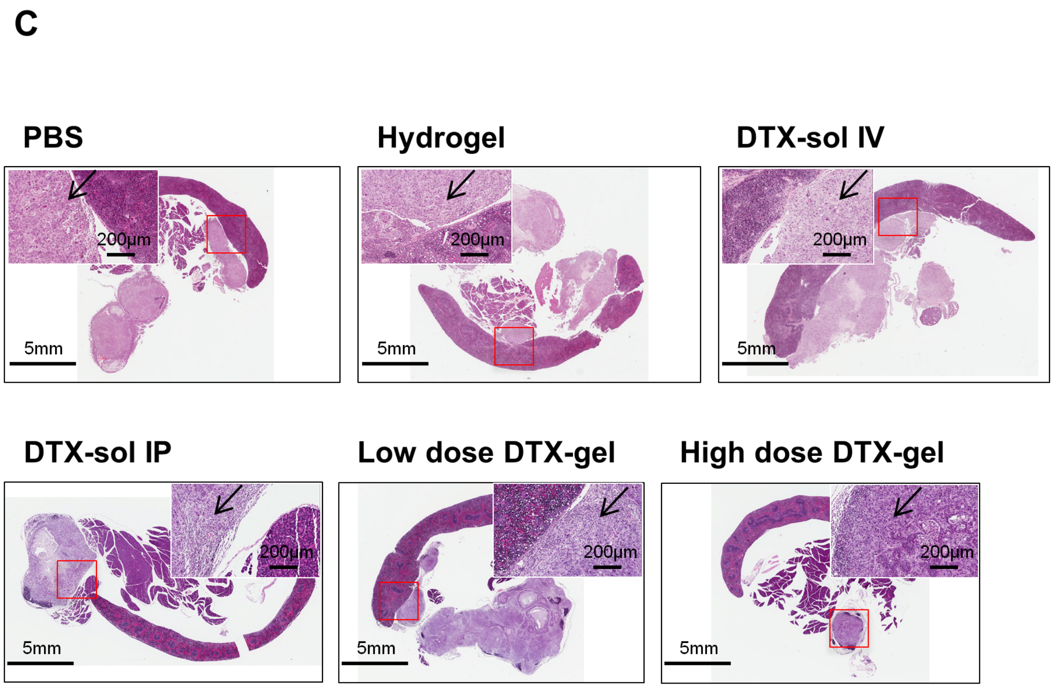

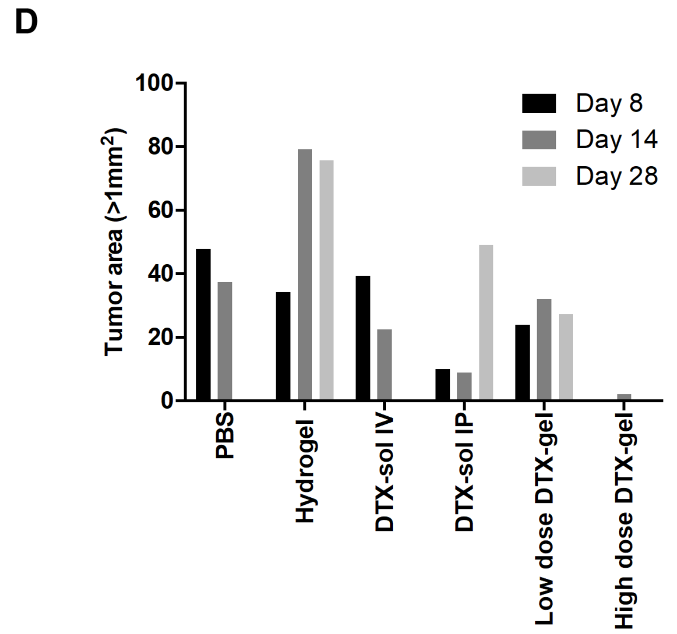

Supplementary Figure 5: Histological examinations of liver, kidney and spleen. **(A)** The tumor masses were found in liver and kidney tissues. The involved liver tumors were found in PBS, hydrogel, DTX-sol IV and low dose DTX-gel groups on day 14, and DTX-sol IP group on day 28. The black bars indicate size of 200 µm, and arrows indicate liver tumors. The tumor masses around kidney were found in PBS, hydrogel, DTX-sol IV and IP groups on 14 days. The black bars indicate 1 mm, and arrows indicate tumor masses around kidney. **(B)** The tumor area (>1 mm^2) was histological calculated in liver and kidney tumor mass. Left side graph indicates liver tumor area and right side graph indicates kidney tumor area on 8, 14 and 28 days, respectively. **(C)** The tumor mass between spleen and pancreas were found in all of mice on 14 days. Black bars of bottom and upper site indicate 5 mm and 200 µm scale, respectablyand arrow indicates tumor mass. **(D)** The tumor area (>1 mm^2) of spleen tumor mass.

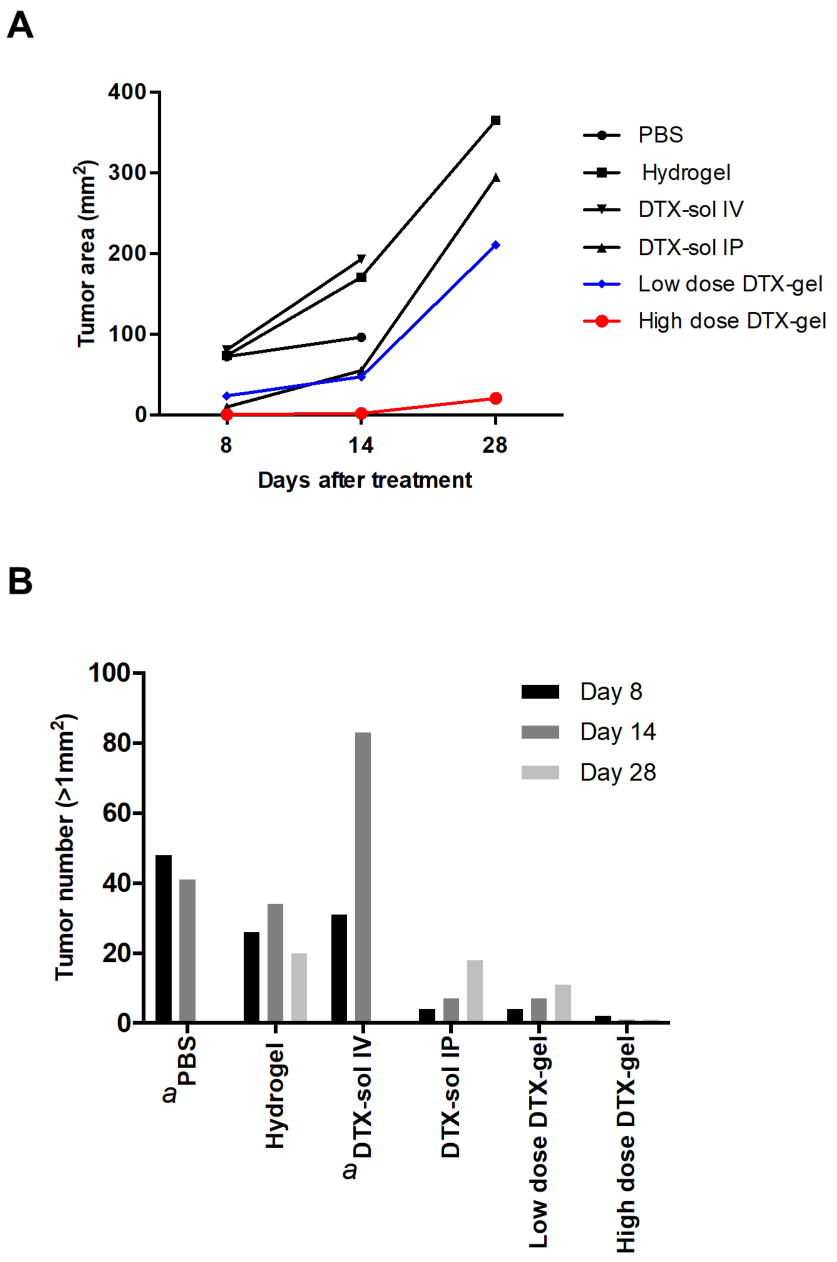

Supplementary Figure 6: Histological examinations of total tumor area and tumor number from metastasized tumors in stomach, kidney, spleen, liver, and mesentery. **(A)** Histologically confirmed tumor area (>1 mm^2) and **(B)** tumor number (>1 mm^2). [a] PBS and DTX-sol IV treated mice were already died before 28 days.

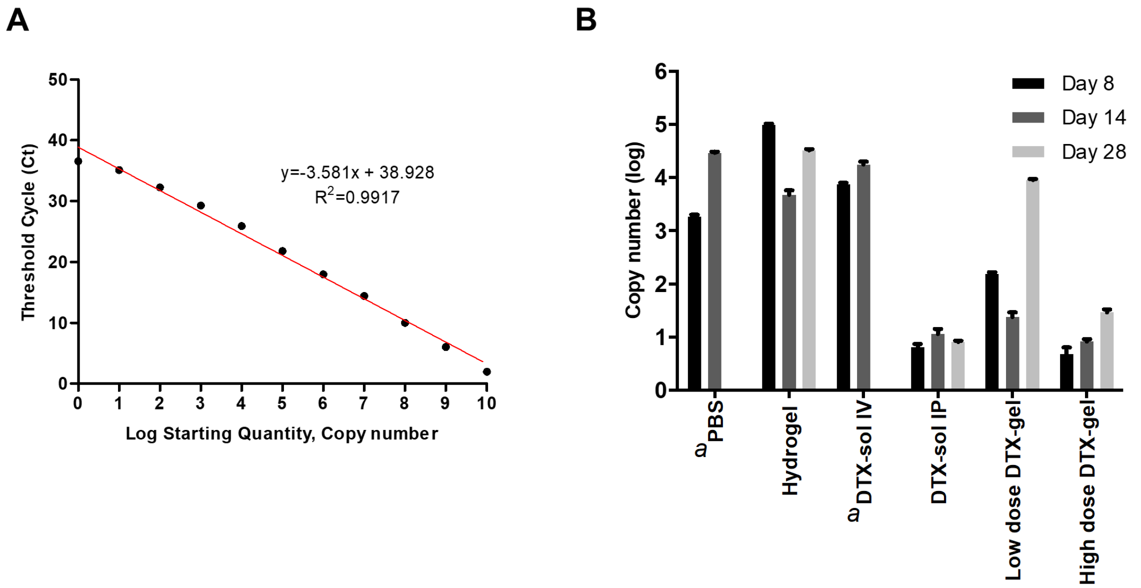

Supplementary Figure 7: CEA level from peritoneal cavity wash samples. CEA level from peritoneal wash samples on 8, 14 and 28 days. **(A)** Standard curve of CEA mRNA and **(B)** CEA copy number for PBS, Hydrogel, DTX-sol IV, DTX-sol IP, Low dose DTX-gel and High dose DTX-gel on 8, 14 and 28 days. [a] PBS and DTX sol IV treated mice were already died before 28 days.

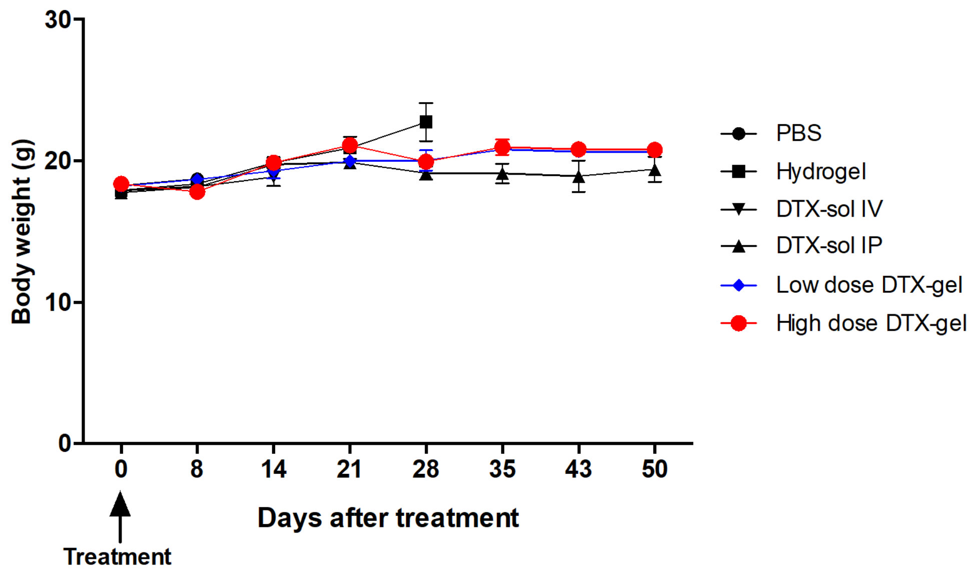

Supplementary Figure 8: Body weight of each mouse after treatments. Body weight was measured each group once a week. The body weight was increased in hydrogel injected group from 21 days due to accumulation of ascites. The low dose and high dose DTX-gel groups similarly maintained their weights until 50 days.
